# Seal upon Physicians Lips No Shield for Unlawful Acts

**Published:** 1897-08

**Authors:** 


**Seal Upon Physicians Lips no Shield for Unlawful Acts.**—In the case of Hauk v. State, where the supreme court of Indiana affirmed a conviction of producing an abortion, the court holds, February 16, 1897, that the rule declared by the statute, which forbids a physician to reveal in evidence matters discovered by him in the course of professional attendance or treatment of a patient, is intended to protect the latter and not to shield one who is charged with perpetrating an unlawful act upon the patient. The statute, it insists, can not be so construed as to permit a party charged with crime to invoke it as a weapon of defense in his own favor instead of its being used as a protection to his victim. This interpretation, in its opinion, accords with reason and is supported by authority. On these grounds the court holds that it was not error to permit the physician who attended the patient in question, at the request of the defendant, at the time of her alleged miscarriage, to give evidence of what he discovered upon an examination of his patient during his attendance, and also the fact that the miscarriage occurred while he was present as her physician. The fact that her death may have resulted from the improper treatment of her physician or otherwise, it further holds, would not operate to defeat the conviction, under the statute, of one who produced her miscarriage by an unlawful antecenent act.—*Journal of the American Medical Association.*

